# Magnitude of road traffic accident related injuries and fatalities in Ethiopia

**DOI:** 10.1371/journal.pone.0202240

**Published:** 2019-01-29

**Authors:** Teferi Abegaz, Samson Gebremedhin

**Affiliations:** 1 School of Public Health, Addis Ababa University, Addis Ababa, Ethiopia; 2 School of Public Health, Hawassa University, Hawassa, Ethiopia; Universitat de Valencia, SPAIN

## Abstract

**Background:**

In many developing countries there is paucity of evidence regarding the epidemiology of road traffic accidents (RTAs). The study determines the rates of injuries and fatalities associated with RTAs in Ethiopia based on the data of a recent national survey.

**Methods:**

The study is based on the secondary data of the Ethiopian Demographic and Health Survey conducted in 2016. The survey collected information about occurrence injuries and accidents including RTAs in the past 12 months among 75,271 members of 16,650 households. Households were selected from nine regions and two city administrations of Ethiopia using stratified cluster sampling procedure.

**Results:**

Of the 75,271 household members enumerated, 123 encountered RTAs in the reference period and rate of RTA-related injury was 163 (95% confidence interval (CI): 136–195) per 100,000 population. Of the 123 causalities, 28 were fatal, making the fatality rate 37 (95% CI: 25–54) per 100,000 population. The RTA-related injuries and fatalities per 100,000 motor vehicles were estimated as 21,681 (95% CI: 18,090–25,938) and 4,922 (95% CI: 3325–7183), respectively. Next to accidental falls, RTAs were the second most common form of accidents and injuries accounting for 22.8% of all such incidents. RTAs contributed to 43.8% of all fatalities secondary to accidents and injuries. Among RTA causalities, 21.9% were drivers, 35.0% were passenger vehicle occupants and 36.0% were vulnerable road users including: motorcyclists (21.0%), pedestrians (12.1%) and cyclists (2.9%). Approximately half (47.1%) of the causalities were between 15–29 years of age and 15.3% were either minors younger than 15 years or seniors older than 64 years of age. Nearly two-thirds (65.0%) of the victims were males.

**Conclusion:**

RTA-related causalities are extremely high in Ethiopia. Male young adults and vulnerable road users are at increased risk of RTAs. There is a urgent need for bringing road safety to the country's public health agenda.

## Background

Road traffic accident (RTA) is a major public health and development challenge. Every year nearly 1.3 million people lose their lives on the road and as many as 50 million others are injured [1]. Globally 17 road fatalities per 100,000 population per annum are reported. RTA is the second leading cause of death in economically active population group of 15–44 years of age; further, more than 75% of RTA casualties occur in this age group [2–4]. In many countries the estimate economic loss due to RTAs is as high as 3% of their gross domestic products [5].

The burden of RTA is disproportionally high in low- and middle-income countries (LMIC) were over 85% fatalities and 90% of disability-adjusted life years lost are reported [3,6,7]. Fatalities secondary to RTAs is at least two-times common in LMIC than in high-income countries. Globally RTA fatalities remain more or less constant since 2007; yet, in many developing countries the rates are increasing [8]. Especially Africa faces the highest annual rate of road fatalities in the world– 27 per 100,000 population [8]. In the next few decades, the problem can even rise due to the ongoing rapid economic growth and increase in motorization in the continent [2,6,9].

Despite the growing burden of RTAs, road safety remains a neglected issue in many developing countries and the health sector has been slow to recognize it as a priority public health problem [8,10]. A large body of evidence suggests that RTAs are easily preventable and many high income countries have successfully reduced the incidence through proven and cost-effective interventions [2,11]. The Sustainable Development Goals (SDGs) Goal-3 sets an ambitious target to halve the global number of fatalities and injuries from RTAs by 2020 [12].

Like many African countries Ethiopia is facing enormous road safety crisis. Each year thousands of road users are killed and the majority of them are economically active population [13]. According to the estimate of the World Health Organization (WHO), in 2013 the prevalence of road traffic fatality in Ethiopia was 25.3 per 100,000 population and the rate is among the highest in the world [8]. Factors contributing to the high incidence of RTAs in Ethiopia include rampant reckless driving behaviors, poor road network, substandard road conditions, failure to enforce traffic laws and poor conditions of vehicles [14].

In many developing countries including Ethiopia there is paucity of evidence regarding the incidence of RTA-related injuries and fatalities. Further, the available estimates based on official reports are likely to underestimate the extent of the problem [15]. In this study, we examined the magnitude of RTA-related injuries and fatalities in Ethiopia based on the secondary data of a nationally representative survey conducted in 2016. We hope, the findings of the study will help to bring forward the agenda of road safety to the attention of public health decision makers. Further, the study will contribute for the evaluation and monitoring of ongoing interventions in the country.

## Methods

### Study design

This study is based on the data of the Ethiopian Demographic and Health Survey (EDHS) conducted in 2016. The DHS is a standard survey that is being carried out regularly in several LMIC including Ethiopia. The DHSs are implemented by the respective national agencies in close collaboration with the DHS program. The DHS gathers nationally representative health and population data and provides updated estimates of key health and demographic indices. The EDHS– 2016 for the first time has collected information on injuries and accidents including RTAs [16].

### Sampling approach

The EDHS 2016, collected information about the occurrence of road traffic and other accidents in the preceding 1 year of the survey among 75,271 members of 16,650 households. As the study was conducted based on readily available data, we did not make priory sample size calculation.

The EDHS– 2016 employed stratified two-stage cluster sampling procedure designed to provide representative sample for multiple health and population indicators at national, place of residence (urban-rural) and sub-national levels (nine geographical regions and two city administrations). Initially, 645 enumeration areas (EAs) (202 in urban areas and 443 in rural areas) were drawn using probability proportional to size (PPS) sampling approach from a complete list of 84,915 EAs defined in the recent 2007 population census. Then in every selected EA, an exhaustive listing of households was made and 28 households were selected using systematic random sampling approach. In the selected households, enumeration of the entire members was made and information about the occurrence of injuries and accidents including RTAs among all household members was collected primarily from the household head or his/her partner [16].

### Data collection

The EDHS data were collected from January to June 2016 using standardized and pretested questionnaires prepared in three major local languages. The data collectors, editors and supervisors received a four-weeks training [16]. Information about the occurrence of injuries and accidents including RTAs was assessed by asking the respondents whether any child or adult in their household was killed or injured in the past 12 months in any accident/injury severe enough that the victim could not carry out their normal activities for at least a day [16]. When such incident was reported, additional information (cause and types of injury, length of injury, whether the injury was fatal or not, and the age and sex characteristics of the victim) was explored.

### Data analysis

The “individual” and “household” datasets of the DHS 2016 survey were downloaded separately from the DHS Program website [17] in SPSS format. Information about the victims was extracted from the “household” dataset, then appended to the “individual” dataset and finaly duplicate observations were removed. In order to accommodate for the complex sampling design employed in the survey, weighted data analysis was employed. Data weights were computed using sampling weights readily provided in the dataset and post-stratification weight developed by the researchers based on the 2016 population size of the nine regions and two city administration of the country [18]. The analyzed dataset is available from: https://dhsprogram.com/data/dataset/Ethiopia_Standard-DHS_2016.cfm?flag=0.

The rates of injuries and fatalities secondary to RTAs are presented per 100,000 population. Further, inconsideration of the 2016 midyear population size of the country and the registered number of motor vehicles in country in the same year, the rates were presented per 100,000 vehicles. Ninety-five percent confidence intervals (95% CI) were estimated for the rates using the binomial CI calculator of the STATA software. Household wealth index, a composite indicator of household wealth status, was determined using Principal component Analysis (PCA) based on the ownership of selected valuable household assets. Association between RTA and basic socio-demographic characteristics (age, sex, place of residence, household wealth index) was determined using Bivariable Binary Logistic Regression analyses and the outputs are provided using Odds Ratio (OR) with the respective 95% CIs. Multivariable logistic regression analysis was not employed because we did not anticipate confounding among independent variables considered in the study.

### Ethical considerations

The EDHS 2016 study was ethically cleared by the National Research Ethics Review Committee, Ministry of Science and Technology of Ethiopia [16]. Data were collected after taking informed consent from the respondents.

## Results

### Socio-demographic characteristics

The EDHS 2016 survey gathered data from 16,650 households across the nine regions and two city administrations of the country. The weighted sample distribution shows 80.4% of the households were drawn from rural areas while the remaining 19.6% were from urban areas. The mean (±SD) age of the respondents was 40.1 (±16.1) years and the majorities (57.1%) were women. More than half of the respondents (56.7%) had no formal education and very few (6.1%) had attained tertiary level of education. The median (inter-quartile range) of the household size was 4 (3–6) (Table 1).

**Table 1:** Socio-demographic characteristics of the respondents, Ethiopian Demographic and Health Survey, 2016.

Respondent’s characteristics (n = 16,650)	Frequency	Percentage
Age in years	2,553	15.3
15–24	4,817	28.9
25–34	3,409	20.5
35–44	2,412	14.5
45–54	1,868	11.2
55–64	1,590	9.5
64+	2,553	15.3
Sex		
Male	7,037	42.3
Female	9,613	57.7
Educational status		
No formal education	9,439	56.7
Primary	4,844	29.1
Secondary	1,357	8.1
Higher	1,010	6.1
Place of residence		
Urban	3,259	19.6
Rural	13,391	80.4
Number of household members		
Less than 4	5,675	34.1
4–5	5,294	31.8
5 or above	5,682	34.1

### Magnitude of road traffic accident related injuries and fatalities

The occurrence of RTAs in the past 12 months of the survey was assessed among 75,271 members of the 16,650 households included in the survey. It was found that 123 road accidents–equivalent to a rate of 163 (95% CI: 136–195) per 100,000 population–occurred in the reference 12 months period. Of the 123 causalities, 28 were fatal injuries, making the road traffic fatality rate 37 (95% CI: 25–54) per 100,000 population.

In addition to RTAs, the survey collected information about the occurrence of other forms of accidents and injuries in the same reference period including violence/assault, burn, accidental fall, drowning and poisoning. The rate of all forms of injuries and accidents including RTAs was 716 (95% CI: 657–779) per 100,000 population. RTAs were the second most common form of accident and injury contributing to 22.8% of all such incidents. The other frequent forms of accidents and injuries were: fall (33.2%), violence/assault (14.1%), burns (9.2%), bite or kick by animals (3.9%), poisoning (2.0%) and drowning (1.5%). Nearly one-in-ten (11.9%) of all the injuries and accidents were fatal. RTAs contributed to 43.8% of all these fatalities.

Among 123 individuals who sustained RTAs in the reference period, 21.9% were drivers and 35.0% were passenger vehicle occupants. Further, 36.0% of the victims were vulnerable road users: motorcyclists (21.0%), pedestrians (12.1%) and cyclists (2.9%). Young adults 15 and 29 years constituted 47.1% of all road accidents; further, 15.3% of the casualties were either children younger than 15 years or seniors older than 64 years. Two-thirds (65.0%) of the victims were males.

Among 95 RTA survivors, the duration that the victim was excluded from engaging in normal activities secondary to the injury was assessed. Nearly half (46.2%) of the causalities were precluded from their usual duties for more than a month (Table 2).

**Table 2:** Characteristics of the road traffic accident victims, Ethiopia, 2016.

Characteristics of the road traffic accident victims (n = 123)	Frequency	Percent
Type of the victim (n = 123)		
Passenger vehicle occupants	43	35.0
Drivers	27	21.9
Motorcyclists	26	21.0
Pedestrian	15	12.1
Cyclists	4	2.9
Others	8	6.9
Sex (n = 123)		
Male	80	65.0
Female	43	35.0
Age in years (n = 123)		
0–14	14	11.5
15–29	58	47.1
30–64	46	37.6
65 years or above	5	3.8
Place of residence (n = 123)		
Urban	37	30.4
Rural	86	69.6
Duration that the RTA survivor was precluded from regular activities due to the injury (n = 95)		
Less than 7 days	15	16.3
Between 8 to 30 days	35	37.3
Between 1 to 6 months	34	36.1
More than 6 months	10	10.1

The rate of RTA related injuries and fatalities provided per 100,000 populations can also be presented per 100,000 vehicles. Accordingly, the RTA related injuries and fatalities per 100,000 motor vehicles were 21,681 (95% CI: 18,090–25,938) and 4,922 (95% CI: 3325–7183), respectively.

### Socio-economic factors associated with RTAs

Comparison of the level of occurrence of RTAs across selected socio-demographic characteristics suggested statistically significant variations by place of residence, age, sex and household wealth index. Urban residents had 2.5 times increased odds of the accident as compared to rural residents. Males were nearly two times more likely to sustain road accidents than females. As compared to children 0–14 years of age, adults aged 15–29, 30–64 and 65 years or above had eight, six and four times increased odds of RTAs, respectively. Similarly, the occurrence of RTAs was more frequent among individuals from better-off households (Table 3).

**Table 3:** Association between selected socio-demographic factors and road traffic accident, Ethiopia, 2016.

Variable	RTA		Odds	Odds ratio (95% CI)
	Yes	No		
Place of residence				
Urban	37	11,351	0.0033	2.45 (1.67–3.60)*
Rural	86	63,797	0.0013	1
Sex				
Male	80	37,132	0.0022	1.97 (1.32–2.76)*
Female	43	38,016	0.0011	1
Age (years)				
0–14	14	35,334	0.0004	1
15–29	58	17,453	0.0033	8.26 (4.62–14.76)*
30–64	46	19,003	0.0024	6.06 (3.34–10.98)*
65 years or above	5	3,332	0.0015	3.51 (1.24–9.96)*
Wealth index (quintile)				
Lowest	14	16,561	0.0008	1
Second	22	14,657	0.0015	1.74 (0.90–3.38)
Middle	13	14,621	0.0009	1.01 (0.48–2.15)
Fourth	29	14,791	0.0020	2.22 (1.18–4.19)*
Highest	45	14,518	0.0031	3.56 (1.97–6.45)*

* Statistically significant association at p-value of 0.05.

## Discussion

This study based on nationally representative and population-based data indicated that the rates of injuries and fatalities secondary to RTAs are exceptionally high in Ethiopia and more than one-third of such accidents occur in vulnerable road users including motorcyclists, pedestrians and cyclists. Further, RTAs are more frequent among males, adults 15–29 years of age, urban residents and individuals from better-off households.

In 2013 WHO estimated that, on yearly basis 25.3 road accident fatalities per 100,000 population occur in Ethiopia [8]. The figure is within the 95% confidence limits of our estimate; 37 (95% CI: 25–54) per 100,000 population. Yet, in addition to sampling variation, the higher point estimate observed in our study can be explained by methodological variations. In this study RTA fatality was assessed by asking the respondent whether any member of the household was killed in the past 12 months due to road accident or not. However WHO counts deaths that exclusively occur within the first 30 days of the accident [8]. Accordingly, the latter is likely to exclude late fatalities. Further, WHO estimates are made based on official national reports and a couple of studies testified that official reports are likely to underestimate the magnitude of RTAs [6,19]. A population-based study in Dar es Salaam, Tanzania found that official reports were only filed for about half of the actual RTAs [19].

While more than two-thirds of all traffic accidents occurred among rural residents, urban residents have an elevated injury rate. This is likely due to the higher volume of traffic and human mobility in urban areas. To the best of our knowledge, no study from a developing country explored the urban–rural variations in the incidence of RTAs. However, unlike our finding, two studies conducted in developed countries suggested that both crash injury and fatality rates are higher in rural than in urban areas [20, 21]. Here it is also important to note that we compared the urban–rural differences in the occurrence of RTAs based on the usual place of residence of the victim, not on the actual location of the incident.

We found that economically active population 15–64 years of age contributed to nearly 85% of the total burden of RTAs. Especially adults 15–29 years take the highest toll. This finding is suggestive of the high loss of productivity associated with road accidents in Ethiopia. A study conducted in Kenya also found that more than three quarters of all road traffic victims were economically productive young adults [4]. Similarly, WHO estimated that adults aged 15–44 years account for nearly half of the global road traffic deaths [5]. This segment of population is usually the breadwinner of the family and they might be expected to travel long distances to fulfill the needs of their families.

Most of the studies conducted so far consistently indicated pedestrians are the main victims of RTAs. In LMIC pedestrians may account up to 75% of the total RTA fatalities [3,6]. However, in this study car occupants were more frequently involved in road crashes. This might be explained by the fact that most of the previous studies were based on institutional data and they were mainly limited to urban settings. In urban areas of low income countries, pedestrians commonly share roads with fast moving vehicles as a result they may disproportionately sustain RTAs. Yet, in our study 80% of participants were rural residents with relatively less exposure to RTA as a pedestrian. A couple of studies have suggested that passengers are at higher risk of sustaining injury in intercity highways [4,22]. In Libya a study based on medical records indicated, due to RTAs vehicle occupants were more often admitted to hospitals than pedestrians do [23].

The study indicated two-thirds of the victims of RTAs are males and they have nearly twofold increased odds of RTAs than females. Previous studies from both developed and developing countries consistently witnessed that males are disproportionately involved in traffic accidents [24–28]. Studies also documented clear variations in the accident involvement and risky driving behaviors between the two genders in favor of females [25,29–31]. Though males are frequently considered as skilled drivers, they often engage in risky behaviors including trespassing speed limits, reckless overtaking, non-use of seatbelts and driving under the influence of alcohol [25,29]. Male drivers also tend to have lower attention, impatience and risk perception than females [25,30]. The more frequent involvement of males in cycling and riding of two-wheeled motor vehicles may also contribute for their disproportionate burden [24]. Further, in developing countries like Ethiopia, females are less engaged in outdoor activities and this may reduce their susceptibility to RTAs.

Very few studies so far measured the incidence of RTAs based on community-based data [32–35]. This might be due to the reason that road crashes are rare events in statistical sense and community-based studies have to enroll unmanageably large number of subjects for estimating the incidence with acceptable precision. A nationwide study in Nepal that surveyed 2,695 subjects, found 2.7% prevalence of RTAs [32]. A study that included 6,000 individuals from Dar es Salaam, Tanzania determined non-fatal RTAs incidence of 32.7 per 1,000 person-years [33]. In Hyderabad city in Southern India, based on the data of more than 10,000 individuals, reported three-years period prevalence of 20.7% for non-fata RTAs [34]. A study conducted in 2008 in Teheran, Iran, based on secondary data of more than 64,000 adults found 17.3 traffic accidents per 1000 population, per year [35]. The rates reported in these studies cannot be directly compared with our finding or against to each other due to variations in operational definitions, study setting and reference periods.

The typical strength of the study is the fact that it availed nationally representative information on the magnitude of both RTA-related injuries and fatalities based on population-based data. Most of the existing estimates only measured incidence of fatalities and they were reliant on official reports which are liable to coverage errors [6,19]. The study also assessed the epidemiological significance of RTAs in comparison with other forms of accidents and injuries. Such information has not been frequently presented in previous undertakings.

Conversely, the following key limitations should be considered while interpreting the findings of the study. Firstly, despite the large sample size we used, rates of RTA-related injuries and fatalities were estimated based on small numbers of accidents. This has undoubtedly affected the precision of the estimates. Further, the smaller numbers of events did not allow us for estimation of rates for narrower age intervals and different geographical regions of the country. Secondly, the DHS only enumerates RTAs that were severe enough to preclude the victim out of their normal activities for at least a day. While the definition is sensible enough to detect moderate injuries, it may overlook minor accidents like soft tissue injuries. Finally, the study only assessed the socio-demographic differentials of RTAs and did not explore more critical risky behaviors of drivers.

## Conclusion

Road accident related injuries and fatalities are exceptionally high in Ethiopia and disproportionally affect the economically productive segment of the population. Further, males, individuals from better-off households and vulnerable road users including motorcyclists, pedestrians and cyclists are at increased risk of RTAs. RTA should be considered as a priority public health problem in Ethiopia and there is an urgent need for bringing road safety to the country's public health agenda. While safety interventions including law enforcements and education should include all road users; implementing interventions targeted at young adults and vulnerable road users are critically need to reduce their disproportionate vulnerability. Experimental studies for identifying effective and cost efficient road safety intervention that are feasible in Ethiopian context are also needed.

## References

[1] Sleet D, Baldwin G, Dellinger A, Dinh-Zarr B. The decade of action for global road safety. J Safety Res. 2011; 42: 147–148. 10.1016/j.jsr.2011.02.001 21569898

[2] Mohan D. Road safety in less motorized environments: Future concerns. Int J Epidemiol. 2002; 31: 527–532. 12055145 10.1093/ije/31.3.527

[3] Nantulya V, Reich M. The neglected epidemic: Road traffic injuries in developing countries. BMJ. 2002; 324: 1139–1141. 12003888 10.1136/bmj.324.7346.1139 PMC1123095

[4] Odero W, Khayesi M, Heda P. Road traffic injuries in Kenya: Magnitude cause and status of intervention. Inj Control Saf Promot. 2003; 10: 53–61. 10.1076/icsp.10.1.53.14103 12772486

[5] World Health Organization. Road traffic injuries: Key facts, 2018. Accessed from: http://www.who.int/news-room/fact-sheets/detail/road-traffic-injuries. Cited 01 July 2018.

[6] Peden M, Scurfiled R, Sleet D, Mohan D, Hyder A, Jarawan E. World report on road traffic injury prevention Geneva: World Health Organization; 2004.

[7] Ameratunga S, Hijar M, Norton R. Road-traffic injuries: Confronting disparities to address a global-health problem. Lancet. 2006; 367: 1533–1540. 10.1016/S0140-6736(06)68654-6 16679167

[8] World Health Organization. Global status report on road safety 2015: Summary. Geneva: WHO; 2018.

[9] Bishai D, Quresh A, James P, Ghaffar A. National road casualties and economic development. Health Economics. 2006; 15: 65–81. 10.1002/hec.1020 16145717

[10] Watkins K. Safe and sustainable roads: An agenda for Rio+20. 2012. Accessed from: http://www.youthforroadsafety.org/uploads/nieuws_bijlagen/rio_20_report_lr.pdf. Accessed on: July 01, 2018.

[11] Elvik R. The stability of long-term trends in the number of traffic fatalities in a sample of highly motorised countries. Accid Anal Prev. 2010; 42: 245–60. 10.1016/j.aap.2009.08.002 19887165

[12] United Nations Development Program. Sustainable Development Goals, 2018. Accessed from: http://www.undp.org/content/undp/en/home/sustainable-development-goals.html. Cited 01 July, 2018.

[13] Ethiopian Federal Police Commission. Road traffic accident report. Addis Ababa: Ethiopian Federal Police Commission; 2017.

[14] Persson A. Road traffic accidents in Ethiopia: Magnitude, causes and possible interventions. Advances in Transportation Studies. 2008; 15: 5–16.

[15] Abegaz T, Berhane Y, Worku A, Assrat A, Assefa A. Road traffic deaths and injuries are under-reported in Ethiopia: A capture-recapture method. PLoS ONE. 2014; 9(7): e103001 10.1371/journal.pone.0103001 25054440 PMC4108419

[16] Central Statistical Agency [Ethiopia] and the DHS Program. Ethiopia: Demographic and Health Survey 2016. Addis Ababa and Rockville: CSA and DHS Program; 2017.

[17] The DHS Program. Data, 2018.Accessed from: https://dhsprogram.com/Data/; Cited June 30, 2018.

[18] Population Census Commission of Ethiopia. Summary and statistical reports of the 2007 population and housing census: Population size by age and sex. Addis Ababa: Population Census Commission; 2008.

[19] Zimmerman K, Mzige AA, Kibatala PL, Museru LM, Guerrero A. Road traffic injury incidence and crash characteristics in Dar es Salaam: A population based study. Accid Anal Prev. 2012; 45: 204–210. 10.1016/j.aap.2011.06.018 22269502

[20] Zwerling C, Peek-Asa C, Whitten P, Choi S, Sprince N, Jones M. Fatal motor vehicle crashes in rural and urban areas: Decomposing rates into contributing factors. Inj Prev. 2005; 11(1): 24–28. 10.1136/ip.2004.005959 15691985 PMC1730169

[21] Brown LH, Khanna A, Hunt RC. Rural vs urban motor vehicle crash death rates: 20 years of FARS data. Prehosp Emerg Care. 2000; 4(1): 7–13. 10634275 10.1080/10903120090941551

[22] Qirjako G, Burazeri G, Hysa B, Roshi E. Factors associated with fatal traffic accidents in Tirana, Albania: Crosssectional study. Croat Med J. 2008; 49: 734–740. 10.3325/cmj.2008.49.734 19090597 PMC2621023

[23] Bodalal Z, Bendardaf R, Ambarek M. A study of a decade of road traffic accidents in Benghazi—Libya: 2001 to 2010. PLoS ONE. 2012; 7(7): e40454 10.1371/journal.pone.0040454 22792332 PMC3394723

[24] Martin JL, Lafont S, Chiron M, Gadegbeku B, Laumon B. Differences between males and females in traffic accident risk in France. Rev Epidemiol Sante Publique. 2004; 52(4): 357–367. 15480293 10.1016/s0398-7620(04)99065-7

[25] Al-Balbissi AH. Role of gender in road accidents. Traffic Inj Prev. 2003; 4(1): 64–73. 10.1080/15389580309857 14522664

[26] World Health Organization. Gender and road traffic injuries, 2002. Accessed from: http://apps.who.int/iris/bitstream/handle/10665/68887/a85576.pdf;jsessionid=0A718DCA8E3D31D0E42963F53BA41005?sequence=1. Cited 03 July, 2018.

[27] Bener A, Crundall D. Role of gender and driver behaviour in road traffic crashes. Int J Crashworthiness. 2008; 13(3): 331–336.

[28] Santamariña-Rubio E K Pérez, Olabarria M, Novoa AM. Gender differences in road traffic injury rate using time travelled as a measure of exposure. Accid Anal Prev. 2014; 65: 1–7. 10.1016/j.aap.2013.11.015 24384384

[29] Baker DR, Clarke S R, Brandt EN Jr. An analysis of factors associated with seat belt use: Prevention opportunities for the medical community. J Okla State Med Assoc. 2000; 93: 496–500. 11077757

[30] Cordellieri P, Baralla F, Ferlazzo F, Sgalla R, Piccardi L, Giannini AM. Gender effects in young road users on road safety attitudes, behaviors and risk perception. Front Psychol. 2016; 7: 1412, 10.3389/fpsyg.2016.01412 PMC5037216 27729877

[31] Glendon AI, Dorn L, Davies DR, Matthews G, Taylor RG. Age and gender differences in perceived accident likelihood and driver competences. Risk Anal. 1996; 16(6): 755–762. 8972107 10.1111/j.1539-6924.1996.tb00826.x

[32] Nepal S, Gupta S, Wong EG, Gurung S, Swaroop M, Kushner AL, et al Burden of road traffic injuries in Nepal: Results of a countrywide population-based survey. Lancet. 2015; 385(S7), 10.1016/S0140-6736(15)60802-9 26313109

[33] Zimmermana K, Mzige AA, Kibatalac PL, Muserud LM, Guerrero A. Road traffic injury incidence and crash characteristics in Dar es Salaam: A population based study. Accid Anal Prev. 2011; 10.1016/j.aap.2011.06.018 22269502

[34] Dandona R, Kumar GA, Ameer A, Ahmed GM, Dandona L. Incidence and burden of road traffic injuries in urban India. Inj Prev. 2008; 14(6): 354–359. 10.1136/ip.2008.019620 19074239 PMC2777413

[35] Sehat M, Naieni KH, Asadi-Lari M, Foroushani AR, Malek-Afzali H. Socioeconomic status and incidence of traffic accidents in Metropolitan Tehran: A population-based study. Int J Prev Med. 2012; 3(3): 181–190. 22448311 PMC3309632

